# Infection Rates and Symptomatic Proportion of SARS-CoV-2 and Influenza in Pediatric Population, China, 2023

**DOI:** 10.3201/eid3009.240065

**Published:** 2024-09

**Authors:** Chao Shi, Yuhe Zhang, Sheng Ye, Jiyang Zhou, Fuyu Zhu, Yumeng Gao, Yan Wang, Bingbing Cong, Shuyu Deng, You Li, Bing Lu, Xin Wang

**Affiliations:** Nanjing Medical University, Nanjing, China (C. Shi, Y. Zhang, S. Ye, F. Zhu, B. Cong, S. Deng, Y. Li, B. Lu, X. Wang);; Wuxi Centre for Disease Control and Prevention, Wuxi, China (C. Shi, J. Zhou, Y. Gao, Y. Wang, B. Lu);; The University of Edinburgh, Edinburgh, Scotland, UK (Y. Li, X. Wang)

**Keywords:** COVID-19, SARS-CoV-2, coronavirus disease, severe acute respiratory syndrome coronavirus 2, viruses, respiratory infections, zoonoses, influenza, pediatric, longitudinal cohort, educational settings, infection rates, symptomatic proportions, environmental virus positivity, circulation patterns, China

## Abstract

We conducted a longitudinal cohort study of SARS-CoV-2 and influenza rates in childcare centers and schools in Wuxi, China, collecting 1,760 environmental samples and 9,214 throat swabs from 593 students (regardless of symptoms) in weekly collections during February–June 2023. We estimated a cumulative infection rate of 124.8 (74 episodes)/1,000 persons for SARS-CoV-2 and 128.2 (76 episodes)/1,000 persons for influenza. The highest SARS-CoV-2 infection rate was in persons 18 years of age, and for influenza, in children 4 years of age. The asymptomatic proportion of SARS-CoV-2 was 59.6% and 66.7% for influenza; SARS-CoV-2 symptomatic proportion was lower in 16–18-year-olds than in 4–6-year-olds. Only samples from frequently touched surface tested positive for SARS-CoV-2 (4/1,052) and influenza (1/1,052). We found asynchronous circulation patterns of SARS-CoV-2 and influenza, similar to trends in national sentinel surveillance. The results support vaccination among pediatric populations and other interventions, such as environmental disinfection in educational settings.

SARS-CoV-2 and influenza have caused substantial health threats. SARS-CoV-2 had caused >0.7 billion reported cases and 6.9 million deaths globally by May 2023, when the World Health Organization (WHO) declared the end of the Public Health Emergency of International Concern for COVID-19 (*1*,*2*). After several large waves during 2020–2022, SARS-CoV-2 continued to circulate at lower levels in 2 subsequent waves in November 2022–February 2023 and April–June 2023, according to the WHO Global Influenza Surveillance and Response System (*3*). Seasonal influenza causes annual epidemics, leading to an annual average of 0.3–0.6 million deaths globally (*4*). Although seasonal influenza declined substantially during the early phase of the COVID-19 pandemic, a resurgence of influenza was seen in December 2021 and afterward; activity gradually increased in 2 waves during December 2022–May 2023 (*3*). 

In China, influenza epidemics primarily associated with influenza virus A(H3N2) were observed during April–December 2022, followed by a major SARS-CoV-2 epidemic during December 2022–February 2023 (*5*). The changes observed after nonpharmaceutical intervention (NPI) requirements were eased might reflect shifts in population-level immunity for SARS-CoV-2 and influenza, as well as changes in characteristics associated with Omicron variant predominance, which raises new questions on the infection extent and age-dependent risk profiles for SARS-CoV-2 and influenza.

Young children and students are commonly considered a priority group for prevention and control of seasonal respiratory viral infections, either because of their high susceptibility to severe outcomes or their role in virus transmission (*6*–*8*). However, the extent of influenza infections among pediatric populations and in educational settings remains unclear because of lack of data, as do the infection rate and age distribution of SARS-CoV-2 after the period of Omicron predominance (*9*–*15*). Reviews conducted before the Omicron period of predominance found low SARS-CoV-2 transmission among pediatric populations and in educational settings, yet many of those data were collected while NPIs were in place (*10*,*11*,*13*). New data quantifying infection rates and variations between age groups are necessary to develop and refine vaccination and control strategies for SARS-CoV-2 and influenza. Furthermore, comparing SARS-CoV-2 with influenza could offer insights for delineating between-virus heterogeneities in transmission, epidemic trajectories, and responses to control measures. Previous studies comparing SARS-CoV-2 and influenza have focused on clinical symptoms and severe outcomes, such as hospitalization and death (*16*–*18*), rather than comparing infection risk profiles of the 2 viruses in the same pediatric population.

Accurately estimating the incidence of respiratory viral infections is challenging because infections are usually partially observed in surveillance of active infections. Symptomatic infections are not fully captured because of lack of systematic testing, and mild and asymptomatic infections are particularly underrepresented because testing has primarily focused on symptomatic persons and those with severe illness. Estimating infection risk profile across subgroups and settings is further complicated by variations in the probability of developing symptoms and severe illness. Although seroprevalence indicates the extent of recent infections, distinguishing between seropositivity caused by natural infections or vaccination becomes difficult after widespread vaccination (*9*). Longitudinal cohort studies with prospective and intense virological testing could provide valuable information in this context. Few longitudinal cohort studies have quantified infection rates of SARS-CoV-2 and seasonal influenza among pediatric populations, and particularly after the Omicron wave and widespread vaccination (*11*,*14*,*15*,*19*–*21*).

Schools and childcare centers are primary settings for daily activities and interactions of the pediatric population, making them key settings for the spread of respiratory viruses within that group. We conducted a prospective and longitudinal cohort study in educational settings in Wuxi, China, investigating infection rates and age-dependent risk profiles of SARS-CoV-2 and influenza among the pediatric population. We report data over a full school term, spanning February–June 2023, corresponding to a period of increased influenza and SARS-CoV-2 activity in China (*22*).

## Methods

### Study Design and Participants

We conducted this cohort study in the city of Wuxi in Jiangsu Province, China. Wuxi spans an area of >4,500 km^2^, including 5 districts, 2 county-level cities, and the Economic Development Zone. Wuxi had a resident population of 7.5 million in 2022, of which ≈1 million persons 4–18 years of age were enrolled in childcare centers and primary and secondary schools.

We conducted the study during a spring/summer school term during February–June 2023. We used a multistage sampling scheme, and first selected 2 childcare centers, 2 primary schools, 2 junior secondary schools, and a high school in rural and urban settings (Appendix Table 1). Assuming a 12%–18% attack rate of SARS-CoV-2 and influenza and a 5% loss to follow-up, we aimed to enroll a total of 595 persons and on average 85 persons per school (*23*,*24*). Assuming a lower attack rate of 5%, the sample size is sufficient to detect a difference of 9.5% or more between groups with 80% power and 5% level of significance. Given the required sample size, we randomly selected 4 classes in each childcare center and 2 classes in each school, and all students in the selected classes were invited to participate in this study. The study received ethics approval from Wuxi Center for Disease Control and Prevention (approval no. 202302). Informed consent or parental permission was obtained for all participants. We conducted analyses anonymously.

### Data Collection and Follow-up

Follow-up was conducted weekly for SARS-CoV-2 and influenza infections, regardless of symptoms. Trained school doctors in each institution collected throat swab samples each week, except during the national holiday on May 1 and at the end of June, when semester exams and summer break gradually began across the institutions. School doctors interviewed persons who tested positive for SARS-CoV-2 or influenza, or the parents of children in childcare centers and primary schools, to collect information on respiratory symptoms during the week after the positive test using a predesigned questionnaire (Appendix Table 2). Information on participants’ sex, age, and COVID-19 and influenza vaccination history were collected using questionnaires and by linking with the local vaccination registry.

We collected environmental samples on the day of respiratory sample collection each week in each school. We collected swab samples from frequently touched surfaces (e.g., handrails, floors) and surfaces of infrequent touch (e.g., lights and ceilings) for viral detection (Appendix).

### Laboratory Methods

Respiratory specimens and environmental samples were placed in viral transport medium and transported at 2–8°C to district Center for Disease Control and Prevention laboratories within 6 hours. They were stored at −80°C until nucleic acid extraction. We extracted viral RNA using nucleic acid diagnostic kits (Sansure Biotech [https://www.sansureglobal.com] for SARS-CoV-2; Jiangsu Bioperfectus [https://www.bioperfectus.com] for influenza). We tested each sample for SARS-CoV-2 and influenza virus within 48 hours of its receipt using real-time reverse transcription PCR (RT-PCR) and the nucleic acid test kit.

### Definitions

SARS-CoV-2 and influenza infections were confirmed when throat swabs tested positive on real-time RT-PCR, regardless of symptoms. Multiple positive test results of throat swabs collected from one person within 28 days were considered 1 episode (*14*). We defined a symptomatic SARS-CoV-2 or influenza episode as a person reporting >1 symptom, including fever (body temperature >38°C), respiratory infection symptoms (cough, sore throat, congested nose, runny nose, or sneezing), or other symptoms (fatigue or vomiting).

### Statistical Analysis

We excluded participants who did not meet the eligibility criteria on sample collection and testing (Appendix). We estimated cumulative infection rates and 95% CIs of SARS-CoV-2 and influenza infections by dividing the number of infection episodes by the number of participants using a Poisson distribution. We used the logistic regression model to investigate factors of infections and symptomatic infections. To depict epidemic dynamics of SARS-CoV-2 and influenza, we estimated weekly virus positivity among participants and environmental positivity using the number of positive test results dividing the number of tests each week, in comparison to the weekly influenza and SARS-CoV-2 positivity in influenza-like illnesses (ILI) using country-level sentinel surveillance data (*22*). To account for variations in the infection rate of the viruses across different types of educational institutions, we calculated the relative intensity of the virus using the number of positive tests each week dividing the total positive test results obtained throughout the study period (*25*). We compared proportions with χ^2^ test or Fisher exact tests, as appropriate. We conducted analyses using R version 4.2.3 (The R Project for Statistical Computing, https://www.r-project.org). We considered a 2-sided p value <0.05 to be statistically significant.

## Results

### Study Participants

**Figure 1:**
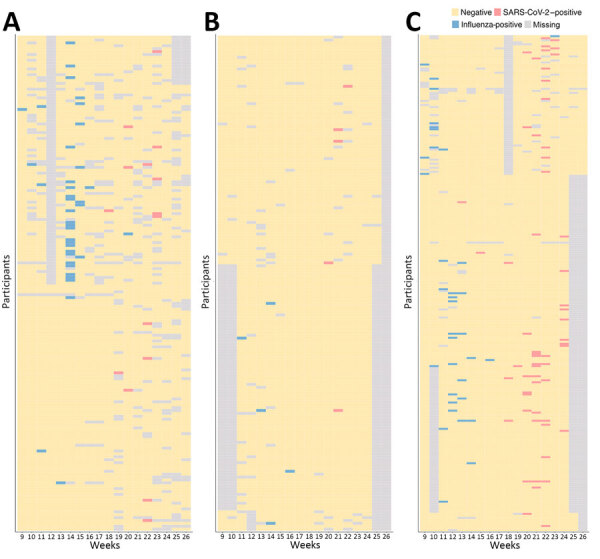
Longitudinal reverse-transcription PCR results of 593 participants by type of educational setting in study of infection rates, symptomatic proportion, and age-dependent risk profiles of SARS-CoV-2 and influenza in pediatric population, China, 2023. A) Childcare centers; B) primary schools; C) secondary schools (both junior secondary schools and high school). Each row shows longitudinal test results of 1 participant. Data are missing if no sample was tested.

We enrolled 666 participants and excluded 73 participants that did not meet the eligibility criteria, yielding 593 (89.0%) participants included in the analysis. During the 18-week study period, we sampled and tested a total of 9,214 throat swabs, and each participant had 15.5 (95% CI 15.4–15.7) RT-PCR tests on average (Figure 1). We collected, on average, 15.3–16.1 respiratory samples across the educational settings and age groups (Appendix Table 4).

### Infection Rates and Age-Dependent Risk Profiles

**Figure 2:**
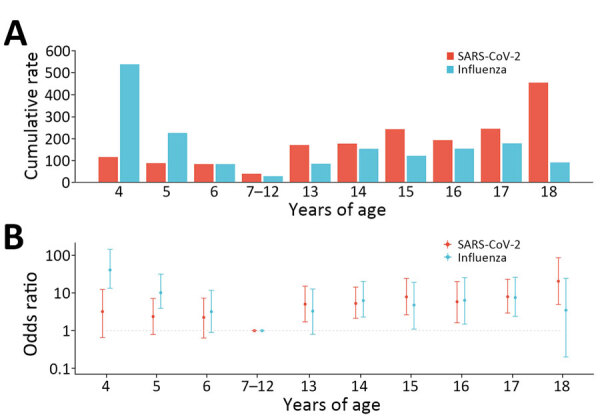
Infection rates and risk for SARS-CoV-2 and influenza infections by narrower age groups in study of infection rates, symptomatic proportion, and age-dependent risk profiles of SARS-CoV-2 and influenza in pediatric population, China, 2023. A) Cumulative infection rates per 1,000 persons; B) odds ratios (log scale) by age. Error bars indicate 95% CIs.

We identified 74 episodes of SARS-CoV-2 infections, yielding a cumulative rate of 124.8 (95% CI 98.0–156.7)/1,000 persons. SARS-CoV-2 infection rates varied significantly between types of educational settings, urban/rural settings, and age groups (Table 1; Figure 2). By types of educational settings, the rate ranged from 40.5 (95% CI 16.3–83.4)/1,000 persons in primary schools to 256.1 (95% CI 158.5–391.5)/1,000 persons in high schools. The infection rate per 1,000 persons was 153.6 (95% CI 115.1–200.9) in urban settings and 84.7 (95% CI 52.4–129.4) in rural settings. The infection rate was highest among persons 18 years of age and lowest in those 7–12 years of age (Figure 2). We found no statistically significant associations between SARS-CoV-2 infection and sex or vaccination history (Table 1).

**Table 1:** Cumulative infection rates of SARS-CoV-2 and influenza and factors of infections among 593 participants in study of infection rates, symptomatic proportion, and age-dependent risk profiles of SARS-CoV-2 and influenza in pediatric population, China, 2023*

Characteristic	No. participants	SARS-CoV-2, n = 74			Influenza, n = 76		p value for SARS-CoV-2 vs. influenza
		Cumulative rates/ 1,000 persons (95% CI)†	Univariate OR (95% CI)		Cumulative rates/ 1,000 persons (95% CI)†	Univariate OR (95% CI)‡	
Overall	593	124.8 (98.0–156.7)	NA		128.2 (101.0–160.4)	NA	0.93
Sex							
F	311	115.8 (81.1–160.3)	Referent		112.5 (78.4–156.5)	Referent	>0.99
M	282	134.8 (95.4–185.0)	1.2 (0.8–2.0)		145.4 (104.3–197.2)	1.4 (0.9–2.3)	0.81
Age group, y§							
4–6	166	90.4 (50.6–149.0)	2.5 (1.0–6.6)		222.9 (156.9–307.2)	10.0 (4.2–29.8)	0.002
7–12	180	38.9 (15.6–80.1)	Referent		27.8 (9.0–64.8)	Referent	0.77
13–15	165	187.9 (127.7–266.7)	5.7 (2.6–14.5)		127.3 (78.8–194.5)	5.1 (2.0–15.6)	0.17
16–18	82	256.1 (158.5–391.5)	8.5 (3.6–22.5)		158.5 (84.4–271.1)	6.6 (2.4–21.2)	0.18
Type of educational setting							
Childcare center	173	86.7 (48.5–143.0)	2.3 (0.9–6.0)		219.7 (155.4–301.5)	11.9 (4.6–40.4)	0.001
Primary school	173	40.5 (16.3–83.4)	Referent		23.1 (6.3–59.2)	Referent	0.54
Junior secondary school	165	187.9 (127.7–266.7)	5.5 (2.5–13.9)		127.3 (78.8–194.5)	6.2 (2.3–21.5)	0.17
High school	82	256.1 (158.5–391.5)	8.2 (3.5–21.6)		158.5 (84.4–271.1)	8.0 (2.7–29.0)	0.18
Urban							
Y	345	153.6 (115.1–200.9)	2.0 (1.2–3.4)		176.8 (135.2–227.1)	3.3 (1.9–6.2)	0.47
N	248	84.7 (52.4–129.4)	Referent		60.5 (33.9–99.8)	Referent	0.39
COVID-19 vaccination history							
Unvaccinated	68	147.1 (70.5–270.4)	Referent		NA	NA	NA
1 dose	19	105.3 (12.7–380.2)	0.7 (0.1–2.9)		NA	NA	NA
2 doses	506	122.5 (93.9–157.1)	0.8 (0.4–1.8)		NA	NA	NA

*ORs were estimated using logistic regression model. NA, not applicable; NE, not estimated; OR, odds ratio. †Cumulative rate and 95% CI indicate the number of episodes dividing the number of participants using Poisson distribution. ‡Influenza vaccination coverage was <1% and thus not included in the analysis for influenza. §The age grouping generally corresponded to different types of educational settings. Most children in childcare centers, primary schools, junior secondary schools, and high schools were 4–6, 7–12, 13–15, and 16–18 years of age.

We identified 76 influenza episodes, yielding a cumulative rate of 128.2 (95% CI 101.0–160.4)/1,000 persons (Table 1). Although the overall infection rate of influenza was similar to that for SARS-CoV-2, the influenza infection rate was higher than SARS-CoV-2 in childcare centers and among children 4–6 years of age (Table 1). Influenza infection rates were associated with types of educational settings, urban or rural settings, and age groups, and the infection rate was highest in childcare centers (219.7/1,000 persons), and lowest in primary schools (23.1/1,000 persons). The infection rate per 1,000 persons was 176.8 (95% CI 135.2–227.1) in urban settings and 60.5 (95% CI 33.9–99.8) in rural settings. By single year of age, the highest influenza infection rate was seen in children 4 years of age (Figure 2). We found no significant association between influenza infection risk and sex. Only 0.5% of the participants had received influenza vaccines in the 2 years before this study, and influenza vaccination history was not assessed in this study.

A total of 6 (8.1%) of the participants infected with SARS-CoV-2 and 5 (6.6%) participants infected with influenza tested positive in 2–3 consecutive weeks. Of those 11 participants, 9 (82%) were 14–15 years of age and 2 were 4–6 years of age.

### Circulation Patterns

**Figure 3:**
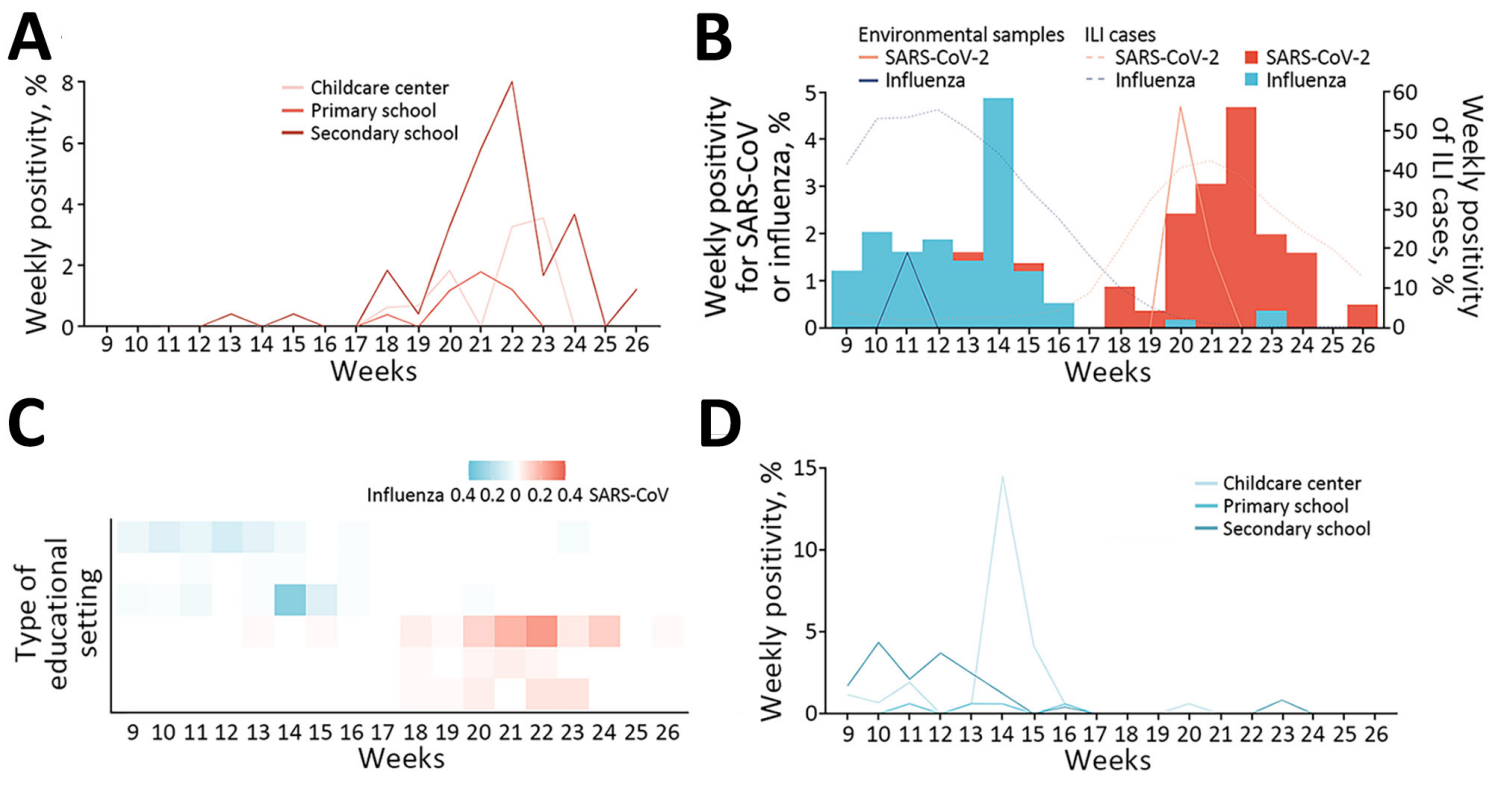
Circulation of SARS-CoV-2 and influenza during the spring/summer school term, February–June 2023, in study of infection rates, symptomatic proportion, and age-dependent risk profiles of SARS-CoV-2 and influenza in pediatric population, China, 2023. The term secondary schools refers to both junior secondary schools and high school. A) Weekly SARS-CoV-2 positivity by type of educational setting. B) Weekly SARS-CoV-2 and influenza positivity among study participants (bars) and from environmental sources (solid lines) and cases of ILI (dotted lines). Scales for the y-axes differ substantially to underscore patterns but do not permit direct comparisons. C) Relative intensity of SARS-CoV-2 and influenza by setting (bottom to top, childcare centers, primary schools, and secondary schools). D) Weekly influenza positivity by type of educational setting. ILI, influenza-like illness.

The study period was from the 9th week through the 26th week of 2023. SARS-CoV-2 infections were sporadically detected during the 13th–15th week in secondary schools; subsequently, infections were identified continuously each week from the 18th through the 26th week (Figure 3, panels A, B). SARS-CoV-2 positivity reached its peak in the 22nd week. The SARS-CoV-2 circulation dynamic varied across types of educational settings; a clearly defined peak and a longer duration of circulation was seen in secondary schools, whereas plateaus and more flattened infection curves were seen in childcare centers and primary schools (Figure 3, panels A, C).

Influenza virus was circulating before SARS-CoV-2 and was detected continuously from the 9th week through the 16th week, reaching its peak in the 14th week (Figure 3, panel B). Sporadic infections were identified during weeks 20–23. Variations in the influenza circulation pattern were found between types of educational settings; influenza infections peaked sharply in childcare centers and more flattened infection curves were seen in secondary schools (Figure 3, panels C, D).

The dynamic of SARS-CoV-2 and influenza positivity generally mirrored that of SARS-CoV-2 and influenza positivity in ILI in the national sentinel surveillance, with some small differences. For instance, the SARS-CoV-2 epidemic peaked in the 22nd week in our study, whereas it peaked in the 21st week at the national level (Figure 3, panel B). Similarly, the influenza epidemic peaked in the 14th week in our study, whereas it peaked during weeks 10–12 at the national level. Moreover, the ILI–influenza positivity at the national level began to increase before the start of the school term.

### Symptomatic Proportion and Symptoms

We collected symptom data for 47 (63.5%) of SARS-CoV-2 episodes and 39 (51.3%) of influenza episodes. Of those, 19 (40.4%) SARS-CoV-2 and 13 (33.3%) influenza episodes were symptomatic. The odds of SARS-CoV-2 symptomatic infection was significantly lower (odds ratio 0.1 [95% CI 0.0–0.8]) among persons 16–18 years of age than for those 4–6 years of age (Table 2). Cough (13/19) was the most frequently reported symptom for SARS-CoV-2, followed by runny nose (9/19) and congested nose (7/19). No participants with symptomatic SARS-CoV-2 infections reported seeking medical care.

**Table 2:** Proportion of symptomatic SARS-CoV-2 and influenza infections, factors of symptomatic infections, and frequency of symptoms in study of infection rates, symptomatic proportion, and age-dependent risk profiles of SARS-CoV-2 and influenza in pediatric population, China, 2023*

Characteristic	SARS-CoV-2			Influenza	
	Symptomatic episodes, n = 47	Univariate OR (95% CI)		Symptomatic episodes, n = 39	Univariate OR† (95% CI)
Overall	19/47 (40.4%)	NA		13/39 (33.3%)	NA
Age group, y					
4–6	4/7 (57.1%)	Referent		11/34 (32.4%)	Referent
7–15	13/24 (54.2%)	0.9 (0.1–4.9)		2/3 (66.7%)	1.8 (0.4–9.6)
16–18	2/16 (12.5%)	0.1 (0.0–0.8)		0/2 (0.0%)	NE
Received >2 doses of COVID-19 vaccine	39/47 (83.0%)	0.3 (0.1–1.6)		NA	NA
Symptom					
Fever	2/19 (10.5%)	NA		6/13 (46.2%)	NA
Cough	13/19 (68.4%)	NA		7/13 (53.8%)	NA
Sore throat	3/19 (15.8%)	NA		0/13 (0.0%)	NA
Congested nose	7/19 (36.8%)	NA		1/13 (7.7%)	NA
Runny nose	9/19 (47.4%)	NA		6/13 (46.2%)	NA
Fatigue	1/19 (5.3%)	NA		0/13 (0.0%)	NA
Vomiting	1/19 (5.3%)	NA		0/13 (0.0%)	NA
Sneezing	0/19 (0.0%)	NA		0/13 (0.0%)	NA
Medical attendance	0/19 (0.0%)	NA		3/13 (23.1%)	NA

*NA, not applicable; NE, not estimated; OR, odds ratio (estimated using logistic regression model). †Influenza vaccination coverage was <5% and thus not included in the analysis for influenza.

The most reported symptoms for influenza were cough (7/13), fever (6/13), and runny nose (6/13) (Table 2). For SARS-CoV-2 and influenza, fever was reported only by participants in childcare centers and primary schools, not in secondary schools (Appendix Table 5). Only 2 participants >16 years of age infected with influenza reported symptom data. In total, 20% of participants with symptomatic influenza infections reported seeking medical care.

### Environmental Samples

We collected and tested a total of 1,052 environmental samples from frequently touched surfaces and 708 samples from infrequently touched surfaces. All of the positive samples (4/1,052) were collected from frequently touched surfaces during weeks 20–21, corresponding to the period of high SARS-CoV-2–positivity among the participants. In contrast, a lower proportion (1/1,052) of environmental samples collected from frequently touched surfaces tested positive for influenza in the 11th week (Figure 3, panel B).

## Discussion

This prospective longitudinal cohort study investigated infection rates, symptomatic proportion, environmental virus positivity, and circulation patterns of SARS-CoV-2 and influenza among pediatric populations in educational settings. Approximately 85% of participants were fully vaccinated with a primary SARS-CoV-2 vaccine, but none received a booster dose before this study; few received influenza vaccination during the 2022–23 influenza season. We estimated, approximately, an overall infection rate of 125 and 128 per 1,000 persons for SARS-CoV-2 and influenza; nearly 60% of participants infected with SARS-CoV-2 and two thirds of participants infected with influenza were asymptomatic. We found different age-dependent infection risk profiles between SARS-CoV-2 and influenza. The highest SARS-CoV-2 infection rate was in persons 18 years of age, and the highest influenza infection rate was in children 4 years of age. A higher risk for symptomatic SARS-CoV-2 infections was seen in the younger group. The 2 viruses were only detected on frequently touched surfaces. We found asynchronous circulation patterns between SARS-CoV-2 and influenza, similar to trends shown in national-level sentinel surveillance data.

Using a longitudinal design and weekly virological testing regardless of symptoms, this study captured asymptomatic and mild infections, offering insights into transmission characteristics of SARS-CoV-2 across age subgroups in comparison with those of influenza. Aligning with previous SARS-CoV-2 systematic reviews that synthesized cohort and contact-tracing studies and a household cohort study from South Africa in which longitudinal virologic testing was conducted, our results of the age-varying SARS-CoV-2 infection risk indicate a higher extent of transmission among secondary school students than younger groups (*11*,*14*,*20*). In contrast to transmission of SARS-CoV-2, our findings suggest a higher extent of influenza transmission in young children than in older groups, consistent with an influenza cohort study in South Africa (*15*). That contrasting age-dependent infection risk profile between SARS-CoV-2 and influenza could be a consequence of varying age-dependent susceptibility and infectiousness between the 2 viruses. Contact tracing and cohort studies suggested higher susceptibility and infectiousness for SARS-CoV-2 in older children (>10 years) than in younger groups, which differs from influenza (*15*,*26*–*30*). A study in Nicaragua showed that persons 10–17 years of age shed SARS-CoV-2 longer than younger children (*26*). Studies in England and South Korea found higher secondary attack rates of SARS-CoV-2 among contacts >10 years of age than among younger children (*27*,*28*). In contrast, a higher secondary attack rate of influenza among younger children was found in studies in the United States and Mongolia (*29*,*30*). The South Africa influenza cohort study showed a higher risk for onward influenza transmission from index case-patients <5 years of age than index case-patients 5–18 years of age (*15*). The variations in influenza infection rates found among children 4 and 6 years of age (Figure 2) indicate that age might influence the spread of influenza within childcare centers. We reported infection rates by educational setting because contact patterns could be strongly influenced by social settings. Our results are generally consistent with a prior US study showing that primary school students tend to have fewer distinct contacts than those in junior secondary schools and high schools (*31*). The infection rates in different educational settings could inform school-based intervention strategies.

A recent meta-analysis found asymptomatic proportions associated with the Omicron variant varied widely from 14% to 57% for all age groups across studies (*32*). However, few studies have reported data for pediatric populations or by granular age groups. Our results (54% for persons 7–15 years of age) are similar to a seroprevalence study in the United States showing that 49% of Omicron infections were symptomatic among children 5–15 years of age; that study found higher symptomatic proportion for Delta infections (*33*). We found a lower probability of symptomatic SARS-CoV-2 infection in persons 16–18 years of age than among young children, which might be partly explained by the gradual development and maturation of human immunity from birth to young adulthood, providing protection against symptomatic infection and more severe illness (*34*). Few studies have reported asymptomatic influenza infections for the pediatric population. Our estimates of symptomatic influenza infections (35% [95% CI 20%–53%] for children 4–15 years of age) appeared slightly lower than that seen in the South Africa influenza cohort study (51% for children 5–12 years of age) (*15*).

A systematic review of studies conducted before the Omicron wave found that fever and cough were the most prevalent symptoms of SARS-CoV-2 and influenza infections among children with a median age of 5–8 years, broadly similar to the symptoms we observed (*16*). Of note, fever was reported only by students in childcare centers and primary schools (those 4–12 years of age) in our study, not by older students (Appendix Table 5). Those findings could indicate possible varying symptom profiles (e.g., fever) across age groups, and the inclusion of older and young children might explain why fever was not the most prevalent symptom in our study.

Only the samples collected from frequently touched surfaces when virus activity was high tested positive for SARS-CoV-2 (4/1,052) and influenza (1/1,052). This finding is consistent with a Hong Kong study conducted in childcare centers and primary schools (*35*). The results suggest that contact by infected persons is a primary cause of environmental contamination in educational settings. Strengthening environmental disinfection could reduce SARS-CoV-2 and influenza transmission in educational settings.

Our results and the data collected by the WHO Global Influenza Surveillance and Response System have shown asynchronous circulation patterns of SARS-CoV-2 and influenza (*3*), which might indicate negative viral interference between the 2 viruses (*36*). Similar results have been indicated by population-level and individual-level epidemiologic data (*37*,*38*) and the low codetection rates of SARS-CoV-2 and influenza in a recent meta-analysis (*39*). A recent model of airway epithelium suggests that SARS-CoV-2 replication could be inhibited by antiviral responses triggered by influenza infections (*40*).

COVID-19 vaccines could prevent infections, thereby reducing virus spread. However, real-life observational data suggest a decline in vaccine effectiveness after 2 doses against infection with the Omicron variant, as well as waning protection over time among children and adolescents (*41*). In total, 80% of participants had received their last SARS-CoV-2 vaccine dose 12 months before, which might explain the lack of significant effect for SARS-CoV-2 vaccination in our study.

The first limitation of our study is that, although no widespread control measures were implemented, some participants might have adopted precautionary behaviors in response to test results, which might have reduced virus spread. Second, variations in the infection rates were observed between schools of the same type, possibly because of differences in virus transmission between the communities where the schools were located. Age-related estimates, which were derived using a generalized linear mixed meta-regression model accounting for between-school variations, were generally comparable to the main analysis, except for the wider CIs of the influenza infection rate for children 4–6 years of age (Appendix Table 6). Nevertheless, the generalizability of our estimates could be limited by the small number of schools included in this study. Third, symptoms of the infections were self-reported; symptom data was lacking among 43% of the infected persons and could cause biases to the estimates of symptomatic proportions (Appendix Table 4). We acknowledge that the estimation of influenza symptomatic proportion for persons 16–18 years of age is not feasible because of the sparce data. Moreover, we did not consider subtypes of SARS-CoV-2 or influenza virus because of lack of data. We reported data collected from students during a school term after a major SARS-CoV-2 epidemic across the country in late 2022 and early 2023; no participants received a booster SARS-CoV-2 vaccine dose before this study, and influenza vaccination coverage was low. We acknowledge that our findings might be context-dependent, influenced by the population’s immunity level related to infection history and vaccination, as well as the implementation of NPIs. Future studies are needed to investigate possible long-term interactions between influenza and SARS-CoV-2.

Overall, we found different age-dependent infection risk profiles between SARS-CoV-2 and influenza. The highest SARS-CoV-2 infection rate was in persons 18 years of age, and for influenza, in children 4 years of age. A higher proportion of symptomatic SARS-CoV-2 infections was seen in young children than in older groups. Our findings can inform both vaccination strategies and other interventions, such as mask wearing, environmental disinfection, and handwashing in educational settings for the control of SARS-CoV-2 and influenza.

Appendix Additional information about infection rates and symptomatic proportion of SARS-CoV-2 and influenza in pediatric population, China, 2023
