# Supplementary material for: Infection Rates and Symptomatic Proportion of SARS-CoV-2 and Influenza in Pediatric Population, China, 2023
Source: Emerg Infect Dis. 2024 Sep;30(9):1809–18. doi: 10.3201/eid3009.240065 (PMC11347007; doi:10.3201/eid3009.240065)
Supplement: Appendix — Additional information about infection rates and symptomatic proportion of SARS-CoV-2 and influenza in pediatric population, China, 2023 [file 24-0065-Techapp-s1.pdf]

Article DOI: https://doi.org/10.3201/eid3009.240065

EID cannot ensure accessibility for supplementary materials supplied by authors. Readers who have difficulty accessing supplementary content should contact the authors for assistance.

Infection Rates and Symptomatic Proportion of SARS-CoV-2 and Influenza in Pediatric Population, China, 2023

Appendix

Data collection of Environmental samples

Environmental sampling was conducted over a surface area of 400 mm^2 (20 mm * 20 mm) for a large surface, such as floors, tables, chairs, countertops, windows, blackboards, water dispensers, baskets, or the entire surface for a small surface, such as handrails, door handles, water-taps, buttons of public facilities, lamp switches, public books.

Eligibility criteria on sample collection and testing

(1)The participants must have had at least one test between the 14th week and the 15th week when influenza activity was high, AND

(2)The participants must have had at least two tests between the 20th and the 23rd week when the SARS-CoV-2 activity was high.

Appendix Table 1. Information on the educational institutions*

Location	School types	Urban/Rural	Age groups	Other
Huishan	Childcare center	Rural	Mostly 4–6 y	NA
Binhu	Childcare center	Urban		NA
Jiangyin	Primary school	Rural	Mostly 7–12 y	NA
Jingkai	Primary school	Urban		NA
Liangxi	Junior secondary school	Urban	Mostly 13–15 y	Separate from the high schools
Yixin	Junior secondary school	Rural		Separate from the high schools
Xishan	High school	Urban	Mostly 16–18 y	Separate from the junior secondary schools; boarding

*NA, not applicable.

Appendix Table 2. Follow-up questionnaire of participants who were tested positive in schools/children care centers.

No. of samples	Grade and class	Name	Date of collection	Results		Records of symptoms	Measures
				SARS-CoV-2	Influenza		
						Your symptoms:	Medical attendance:
						❑Fever	❑Yes
						❑Cough	❑No
						❑Sore throat	Date of medical attendance:
						❑Congested nose	Healthcare institution:
						❑Runny nose	Diagnosis:
						❑Fatigue	Treatment and duration:___
						❑Vomiting	Currently recovered:
						❑Sneezing	❑Yes
						❑Else: ______	❑No
						Date of symptom onset (YYYY/MM/DD)__	Treatment and duration: ________

Appendix Table 3. Surfaces selected for environmental sample collection in different types of educational settings.

Surface type	Educational setting	Items
Frequently touched surfaces	Childcare center	Handrails of stairs and slides
		Door handles, countertops, water-taps, floors and cleaning supplies in toilets
		Floors, tables and chairs of illnesses, cleaning supplies, water dispensers in classes
		Toy storage baskets
		Public books
	Primary and secondary school	Handrails of stairs
		Door handles, countertops, water-taps, floors and cleaning supplies in toilets
		Floors, tables and chairs of illnesses, cleaning supplies, water dispensers and lamp switches in classes
		Seat armrest and tabletop in restaurant
		Buttons of public facilities
Surfaces of infrequent touch	All types of educational settings	Classroom windows and other items in high position, blackboards, lighting fixtures
		High position items in the bathroom, suspended ceilings
		Floors of stairs

Appendix Table 4. Collection of respiratory samples and data missingness on symptoms.

Characteristic	Mean no. of samples (95% CI)	Complete data on SARS-CoV-2 symptomaticity, n (%)	Complete data on influenza symptomaticity, n (%)
Sex			
F	15.6 (15.4–15.8)	23/36 (63.9%)	19/35 (54.3%)
M	15.5 (15.3–15.6)	24/38 (63.2%)	20/41 (48.8%)
Age, y			
4–6	15.4 (15.1–15.7)	7/15 (46.7%)	34/37 (91.9%)
7–12	15.5 (15.3–15.7)	6/7 (85.7%)	1/5 (20.0%)
13–15	15.5 (15.3–15.6)	18/31 (58.1%)	2/21 (9.5%)
16–18	16.1 (15.8–16.4)	16/21 (76.2%)	2/13 (15.4%)
Type of educational setting			
Childcare center	15.3 (15.0–15.6)	7/15 (46.7%)	35/38 (92.1%)
Primary school	15.6 (15.5–15.8)	6/7 (85.7%)	0/4 (0.0%)
Junior secondary school	15.5 (15.3–15.6)	18/31 (58.1%)	2/21 (9.5%)
High school	16.1 (15.8–16.4)	16/21 (76.2%)	2/13 (15.4%)

Appendix Table 5. SARS-CoV-2 and influenza symptoms, by types of educational settings.

Symptom	SARS-CoV-2, n = 19		Influenza, n = 13	
	Childcare center and primary school, n = 6	Secondary school, n = 13	Childcare center and primary school, n = 11	Secondary school, n = 2
Fever	2	0	6	0
Cough	5	8	5	2
Sore throat	1	2	0	0
Congested nose	0	7	0	1
Runny nose	0	9	4	2
Fatigue	0	1	0	0
Vomiting	1	0	0	0
Sneezing	0	0	0	0

Appendix Table 6. Sensitivity analyses of influenza and SARS-CoV-2 infection rates between age groups using the generalized linear mixed meta-regression model.

Age, y	SARS-CoV-2		Influenza	
	Cumulative rates per 1,000 persons* (95% CI)	Univariate OR† (95% CI)	Cumulative rates per 1,000 persons* (95% CI)	Univariate OR† (95% CI)
4–6	86.8 (52.9–138.8)	2.3 (0.9–5.7)	151.7 (49.3–381.5)	10.2 (1.4–74.2)
7–12	40.5 (19.4–82.4)	1 (Referent)	17.3 (3.5–8.1)	1 (Referent)
13–15	187.9 (135.3–254.8)	5.5 (2.3–12.9)	121.4 (39.7–315.9)	7.8 (1.1–58.3)
16–18	256.1 (173.3–361.1)	8.2 (3.3–20.2)	154.9 (33.0–496.0)	10.4 (1.0–106.4)

*Cumulative rate was estimated using generalized linear mixed models.
†OR, odds ratio, estimated using generalized linear mixed models.

Appendix Table 7. OR estimates of SARS-CoV-2 infections associated with age groups and educational settings after adjusting for SARS-CoV-2 vaccination status.

Characteristic	Participants (N)	SARS-CoV-2 (74 episodes / participants)	
		Cumulative rates per 1,000 persons* (95% CI)	Multivariate adjusted OR† (95% CI)
Overall	593	124.85.6 (989.0–156.77.2)	NA
Age, y			
4–6	166	90.4 (50.6–149.0)	2.1 (0.8–5.7)
7–12	180	38.9 (15.6–80.1)	1 (Referent)
13–15	165	187.9 (127.7–266.7)	6.0 (2.7–15.4)
16–18	82	256.1 (158.5–391.5)	9.4 (3.9–25.0)
Type of educational settings			
Childcare center	173	86.7 (48.5–143.0)	1.9 (0.8–5.2)
Primary school	173	40.5 (16.3–83.4)	1 (Referent)
Junior secondary school	165	187.9 (127.7–266.7)	5.8 (2.6–14.7)
High school	82	256.1 (158.5–391.5)	8.9 (3.7–23.9)

*Cumulative rate and 95% CIs: the number of episodes dividing the number of participants using Poisson distribution. NA, not applicable.
†OR, odds ratio, estimated using multivariate logistic regression model adjusting for SARS-CoV-2 vaccination status (2 doses; 0–1 dose).
